# Human Milk Warming Temperatures Using a Simulation of Currently Available Storage and Warming Methods

**DOI:** 10.1371/journal.pone.0128806

**Published:** 2015-06-10

**Authors:** Sharron Bransburg-Zabary, Alexander Virozub, Francis B. Mimouni

**Affiliations:** 1 nanobébé LTD, Tel-Aviv 7239, Israel; 2 Department of Chemical Engineering, Technion, Haifa 32000, Israel; 3 Shaare-Tzedek Medical Center, Jerusalem, Israel; Centre Hospitalier Universitaire Vaudois, FRANCE

## Abstract

Human milk handling guidelines are very demanding, based upon solid scientific evidence that handling methods can make a real difference in infant health and nutrition. Indeed, properly stored milk maintains many of its unique qualities and continues to be the second and third best infant feeding alternatives, much superior to artificial feeding. Container type and shape, mode of steering, amount of air exposure and storage temperature may adversely affect milk stability and composition. Heating above physiological temperatures significantly impacts nutritional and immunological properties of milk. In spite of this knowledge, there are no strict guidelines regarding milk warming. Human milk is often heated in electrical-based bottle warmers that can exceed 80°C, a temperature at which many beneficial human milk properties disappear. High temperatures can also induce fat profile variations as compared with fresh human milk. In this manuscript we estimate the amount of damage due to overheating during warming using a heat flow simulation of a regular water based bottle warmer. To do so, we carried out a series of warming simulations which provided us with dynamic temperature fields within bottled milk. We simulated the use of a hot water-bath at 80°C to heat bottled refrigerated milk (60ml and 178 ml) to demonstrate that large milk portions are overheated (above 40°C). It seems that the contemporary storage method (upright feeding tool, i.e. bottle) and bottle warming device, are not optimize to preserve the unique properties of human milk. Health workers and parents should be aware of this problem especially when it relates to sick neonates and preemies that cannot be directly fed at the breast.

## Introduction

In 2012, the American Academy of Pediatrics (AAP) reaffirmed its recommendation that breastfeeding and human milk (HM) are the normative standards for infant feeding and nutrition. Consequently, exclusive breastfeeding is recommended for about 6 months, followed by continued breastfeeding as complementary foods are introduced [1]. The AAP also added that infant nutrition should be considered a public health issue and not only a lifestyle choice. In other words, formula feeding exposes babies to risks that breastfed babies do not face [1,2].

Human milk is a fresh, living nourishment containing many antioxidant, antibacterial, prebiotic, probiotic, and immune-boosting properties in addition to proteins, essential fats, enzymes, hormones, etc, many of them are uniquely human-coded. Whenever nursing or immediately expressed milk is not available [3], properly stored human milk continues to be the gold standard for infant feeding, superior to artificial feeding [1,4,5], and allows for provision of safe and adequate nutrition. However, like any other living tissue and liquid, HM is sensitive to the effects of temperature [6–12], and some nutrients and bioactive properties may be affected by storage conditions. In particular many enzymes already present in HM initiate milk digestion before milk reaches the intestine, allowing the human infant to rely upon a number of compensatory systems. Thus adequate digestion may be contributed to through maternal enzymes obtained via breastfeeding [6]. For these enzymes to be active require that they do not undergo any temperature-induced denaturation. Thus it becomes vital to make sure that during the process of HM heating to not reach critical temperatures that might lead to denaturation of these bioactive proteins and cause their inactivation. Some studies have shown that during heating of HM, proteolysis increases with increasing temperature and is detectable after 24h at 38°C activity [11]. Digestive enzymes, lipase and amylase are stable for 24 hours at temperatures below 25°C [8] but lose progressively their activity when HM is heated to temperatures of 40–55°C [13].

During heating, not only the temperature that HM may reach is important for enzyme activity, but also the time of exposure to heat is critical. Indeed, when HM is stored at body temperature (38°C), lipolysis is rapid, leading to a 440% rise of free fatty acids (FFAs) content over baseline concentrations within an hour [13]. Another study [14] reported that free fatty acids in human milk increase over time even when stored at 25°C. Such a rise in FFA concentrations is not negligible when we know that such FFAs are cytotoxic and may theoretically lead to cellular damage [15]. Even when HM is stored at 25°C, there is an increase of FFAs concentrations over time [12]. An additional issue related to the integrity of HM is that if damage occurs to HM, it may affect adversely the intestinal microbiome [16] while it is known that the intestinal microbiota play important roles in human neonates, even in the normal development of the brain [17].

HM handling was investigated initially because of the need of HM banks to provide milk of donors. Issues related to storage are also important for mothers who collect their own milk for later feeding. Container type and shape, amount of air exposure and storage temperature may adversely affect milk stability and composition. However, only a few studies have been conducted over the last 30 years that reported the impact of the warming process upon the bio-active value of human milk. Many studies have concluded that microwave ovens or very hot water should not be used to rewarm milk, as the damage due to overheating can be substantial.

The North America Human Milk Banking Association (HMBANA) [18] advocates warming feeds to body temperature for premature infants, particularly those at risk for necrotizing enterocolitis (NEC). For term infants, feedings may be given at body temperature, room temperature or straight from the refrigerator. Several studies were done, in attempts to determine at what temperature the food should actually delivered, at a temperature range from 21.8°C to 46.4°C [19]. It is believed that HM should be warmed to body temperature before its use, in particular in small preterm infants, because of greater feeding tolerance [20] while when HM is overheated, fat absorption may be reduced by about a third [21]. So underwarming may lead to effects on digestion and/or body temperature of the infant. When overwarming may be as detrimental as under warming as it may lead to deterioration of some HM benefits.

Warming may occur in a variety of methods. Clear recommendations have been adopted regarding avoidance of microwave warming [18]. In an attempt to avoid milk overheating, past recommendations have included a preference for warming under “running water”. This is not usually practical in the real world, though. The length of time required to thaw or warm a feeding to an adequate temperature is an unrealistic time for the nurse to spend. The 2011 HMBANA guidelines [18,22] recommends specific processes with rationale regarding the safe handling of human milk guidelines for warming human milk for feeding. They include warming individual feedings in a container of warm water or under running warm water and protecting the container from non-sterile water while noting that communal warming systems may not have clean water. However, both in Neonatal Intensive Care Units (NICU's) and home settings, human milk is often thawed and warmed through a variety of methods not always in compliance with HMBANA recommendations. An additional warming and thawing option is a waterless bottle warmer. Yet, these devices are expensive and are not commonly seen both in the home and NICU settings. Thus, HM is often heated in cups of warm-to-hot tap water, or in electrical water-based bottle warmers. Electrical-based bottle warmers can reach 87°C, well above the HMBANA recommendation of 40°C [18,22].

Warming milk with hot water involves a complex interplay of parameters associated with the milk (volume, initial temperature and thermo-physical properties), its container (geometry and thermo-physical properties) and water (temperature). There is no assurance that in such circumstances milk is heated in a homogeneous manner. Theoretically, within the same bottle, there might be zones of overheating and zones of underheating. The purpose of this study was to use a heat flow simulation model in order to estimate the amount of damage to human milk due to overheating during warming using a standard bottle warmer. These simulations can provide heating gradient profiling of HM stored in an infant bottle and enable to assess the fraction and dynamics of overheated milk. We hypothesized that HM warming in a standard baby bottle, using a standard water based bottle warmer is highly non-homogeneous and may lead to local temperatures within the milk that may be detrimental to milk biological properties.

## Materials and Methods

**Fig 1:**
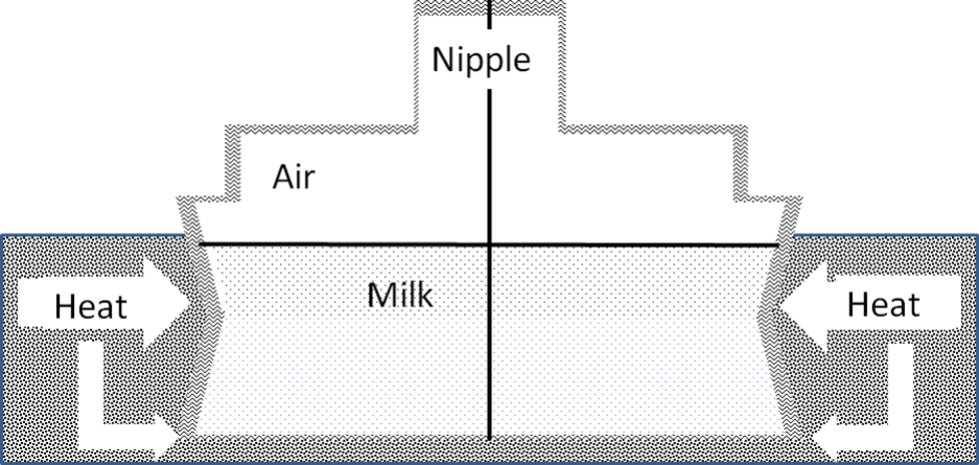
Water bottle warmer model. The model includes milk volume (60 ml or 180ml) indicated as spotted area and upper part filled with air. The bottle height is either 49 mm or 140mm, including the nipple, with total bottle volume either 107 ml or 324ml. The plastic interface (bottle) is indicated in waves, the milk volume is indicated in dots and the heat bath of water is indicated in stripes.

We computationally analyzed heat transfer in a water based electric-bottle warmer, where the emphasis was placed on simulating warming of initially refrigerated milk (4°C) to a “ready to feed” temperature (using the dimensions and geometric spatial properties of the Avent-Philips bottle warmer, Philips International, Amsterdam, The Netherlands). For this purpose, we used the ANSYS-FLUENT software, (ANSYS, Inc. Pennsylvania, USA). A schematic of the model system, assumed to be axially-symmetric and involving surrounding heating water, bottle, milk, air (above the milk) and nipple, is depicted in Fig 1. Operating parameters and relevant physical properties are given in Table 1. For the sake of simplicity, all bottle and nipple properties were assumed identical and their plastic is a generic plastic. This means that these are just generic plastic properties and does not imply that we are assuming the bottle and material are made from this specific material

**Table 1:** Properties and parameters.

Name	Symbol	Value	Comment	Source
**Heat capacity:**	C_p_			[43]
Bottle and nipple	C_p_	1800 J/(Kg K)	Estimated	[44]
Milk	C_p_	3930 J/(Kg K)	Estimated	[45]
Water	C_p_	4182 J/(Kg K)		[45]
air	C_p_	1006 J/(Kg K)		
**Density:**	ρ			
Bottle and nipple	ρ	900 Kg/m^3^	Estimated	[43]
Milk	ρ	1030 Kg/m^3^	Estimated	[46]
Water	ρ	998.2 Kg/m^3^		[45]
air	ρ	1.225 Kg/m^3^		[45]
**Thermal conductivity:**	k			
Bottle and nipple	k	0.2 W/(m K)	Estimated	[47]
Milk	k	0.5369 W/(m K)	Estimated	[48]
Water	k	0.6 W/(m K)		[45]
air	k	0.0242 W/(m K)		[49]
**Thermal expansion coefficient:**	β			
Milk	β	8 X 10^−4^ K^-1^	Estimated	[50] [45]
Water	β	2.07 X 10^−4^ K^-1^		
air	β	3.43 X 10^−3^ K^-1^	Ideal gas at 18°C	
**Viscosity:**	μ			
Milk	μ	3 X 10^−3^ Pa s	Estimated	[51]
Water	μ	1 X 10^−3^ Pa s		[45]
air	μ	1.789 X 10^−5^ Pa s		[45]
**Heat transfer coefficient:**	h	10.45 W/(m^2^ K)	Estimated	[52]
**Milk volume:**				
Bottle 1		60 ml		
Bottle 2		178 ml		
**Bottle height (with nipple):**				
Bottle 1		49 mm		
Bottle 2		140 mm		
**Total bottle volume:**				
Bottle 1		107 ml		
Bottle 2		324 ml		

Our analysis was based on solving relevant equations for heat transport and fluid flow in the various domains. Within all phases we solved the equation for change of temperature based on an assumption of constant thermal conductivity,

$$\frac{\partial T}{\partial t}+v\cdot \nabla T=\alpha_{i}\nabla^{2}T$$ (1)

where T, t and **v** are the temperature, time and velocity vector respectively. The thermal diffusivity of each of the domains is represented by α_i_ where i is an index identifying each of the domains (water, bottle, milk, etc.). It is important to note that the second term on the left hand side of this equation, representing thermal convection, was removed when applying the equation within the solid domains. Within the fluid phases we had to account for flow. This was achieved by solving for the Navier Stokes equation for Newtonian fluids, with the Boussinesq approximation for thermal compressibility, coupled with the continuity equation, which are respectively given by,

$$\rho_{i}\left(\frac{\partial v}{\partial t}+v\cdot \nabla v\right)=-\nabla P+\mu_{i}\nabla^{2}v-\rho_{i}\beta_{i}\left(T-T_{0}\right)g\;,$$ (2)

and

∇⋅v=0, (3)

where ρ_i_ is the density of fluid "i", P is the deviatoric pressure, μ _i_ is the viscosity of fluid "i", β_i_ is the thermal expansion coefficient of fluid "i",**g** is the gravity vector and T_0_ is the characteristic (operating) temperature.

The initial and boundary conditions for the above model equations stem from the logical state of affairs in the real system. Initially, the bottle, nipple, milk and air were all at the refrigerator's temperature (4°C) while the surrounding bath's water temperature was set to 80°C (equivalent to the fastest option of the specific Avent-Philips bottle warmer that we used as a model). All fluid/solid boundaries were assumed to support no-slip and no-penetration conditions for flow, while liquid/gas interfaces were assumed to support no-penetration and no-shear-stress conditions. Temperatures and heat fluxes were assumed to be continuous across all internal boundaries between the different phases. In an effort to simulate the operation of the temperature-controlled heater, the external boundaries of the water in the heater (not including the water/air interface), as well as the outside of the bottom of the bottle, were kept at a time-independent temperature of 80°C. All other external boundaries were assumed to lose heat to the environment via a Newton's law of cooling convective heat flux.

As stated earlier, the Finite-Volume based ANSYS-FLUENT software (ANSYS, Inc. Pennsylvania, USA) was used to solve the relevant equations, using four node quadrilateral elements, where all calculations presented here involved a total of 4293 elements. The transient formulation involved a second order implicit scheme where a time step of either 0.1 or 0.2 seconds was used in the calculations shown in this manuscript. Further refinement of both space and time resulted in insignificant changes in the calculated thermal fields in all the domains. In this calculation average temperatures are depicted, without calculations of standard deviations that have no special meaning/advantage since the importance is on weighing the temperature by the actual mass of the fluid. A better measure is the percentage of milk volume above the maximum allowed temperature (40°C), the temperature that is considered as the upper desired limit [18] in the flowing system that the milk flows into a region of high temperature and then flows out again.

## Results and Discussion

We tested scenarios involving warming of two refrigerated milk volumes (see Table 1) placed in bottles schematically described in Fig 1. As described above, a cold bottle (4°C) was brought into contact with hot water (initially at 80°C). The action of the heater was simulated by keeping the sides and bottom of the water volume (as well as the bottom of the bottle) at 80°C throughout the simulation. The top of the system was assumed to exchange heat with the air present in the nipple at ambient temperature.

**Fig 2:**
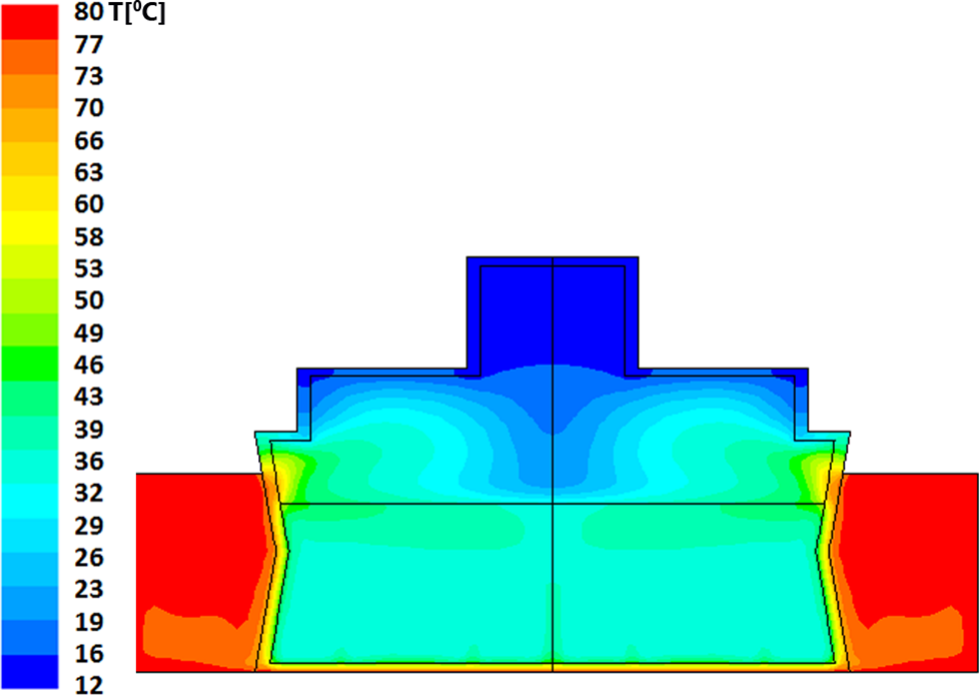
Small volume warming simulation after 150 seconds. The dominant and average temperature is 36°C. There are islets of higher temperature in the upper side of the milk volume below its contact with the air where the maximal temperature reaches 48°C. The milk volume in the temperature range of 39–49°C is approximately 20% of the total volume. The minimal temperature of the milk is 33°C.

**Fig 3:**
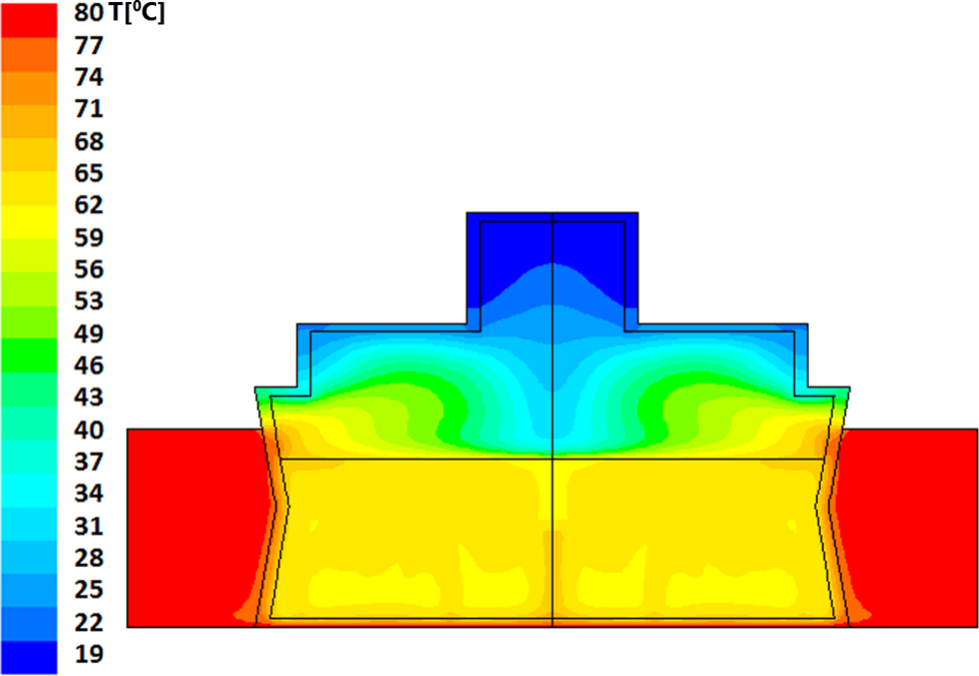
Small volume warming simulation after 420 seconds. The dominant and average temperature is 63°C, with small islets of 60°C and thin islets of 69°C located at the upper interface with the water bath. The minimal temperature of the milk is 60°C.

**Fig 4:**
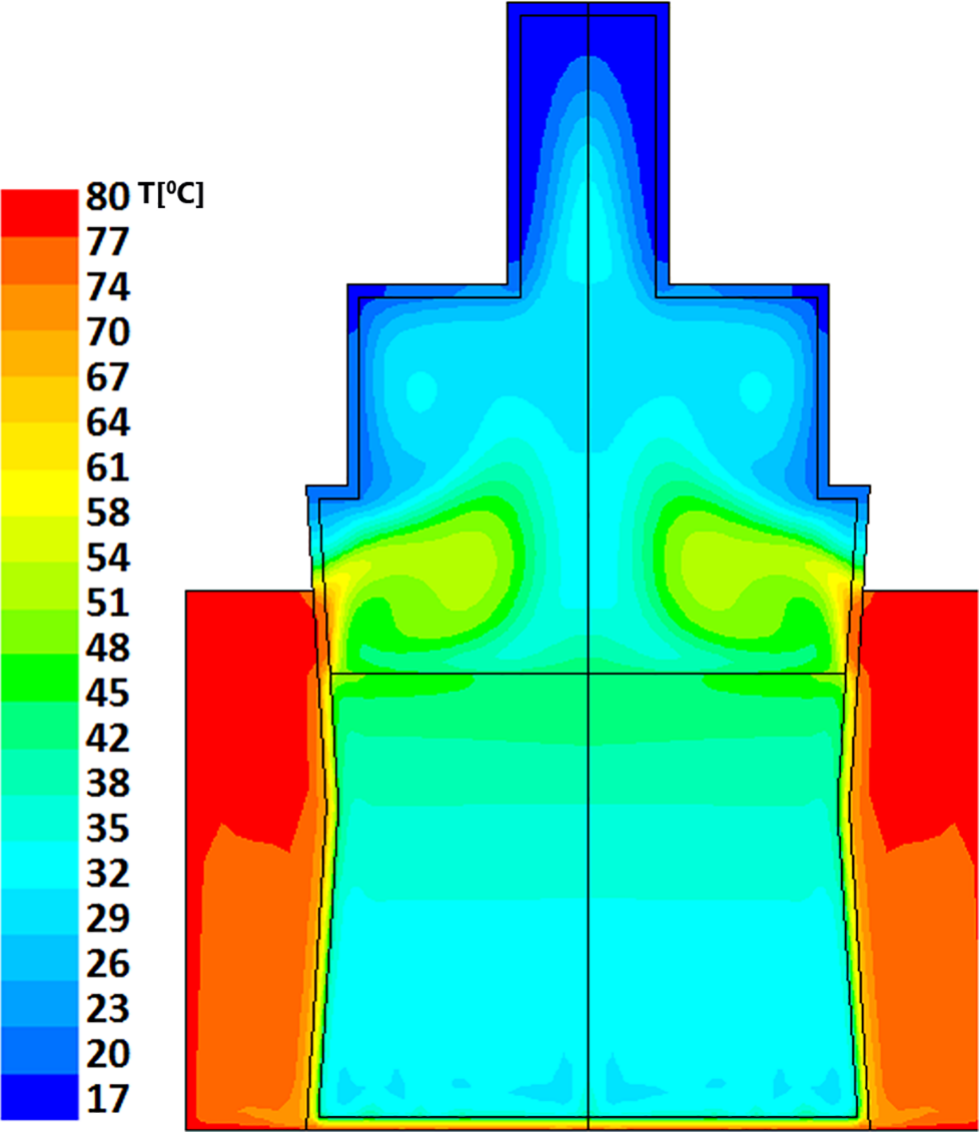
Large volume warming simulation after 300 seconds. The dominant and average temperature is 36°C with layers of higher temperature up to the layer just below the contact with the air where the maximal temperature reaches 53°C. The milk volume having temperature of 40–53°C is approximately 25% of the total volume. The minimal temperature of the milk is 32°C.

**Fig 5:**
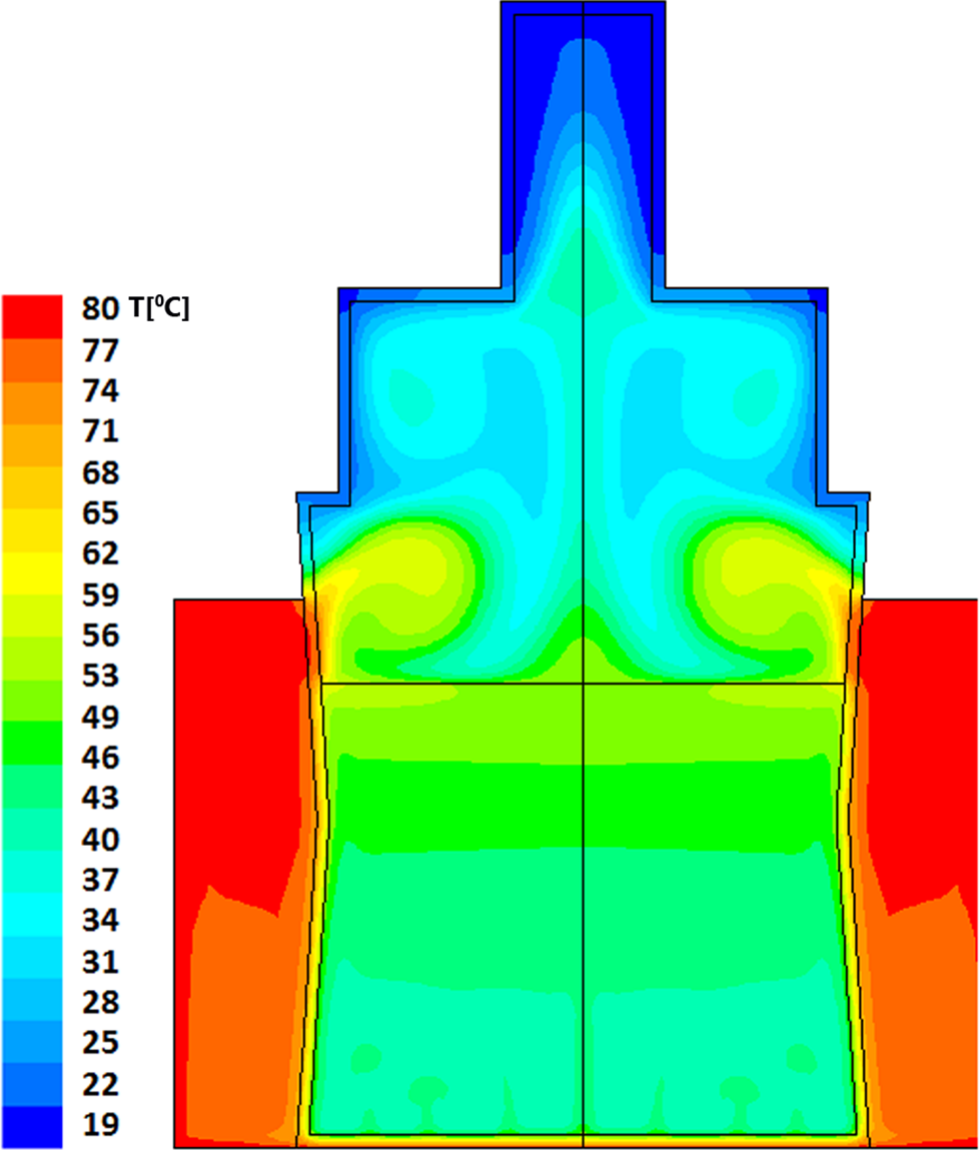
Large volume warming simulation after 420 seconds. The average temperature is 46°C with large islets of 49°C and small islets of 59°C that are located in the upper interface with the water bath. The temperature of the entire milk volume is above 42°C which is just above the recommended heating temperature (40°C).

**Fig 6:**
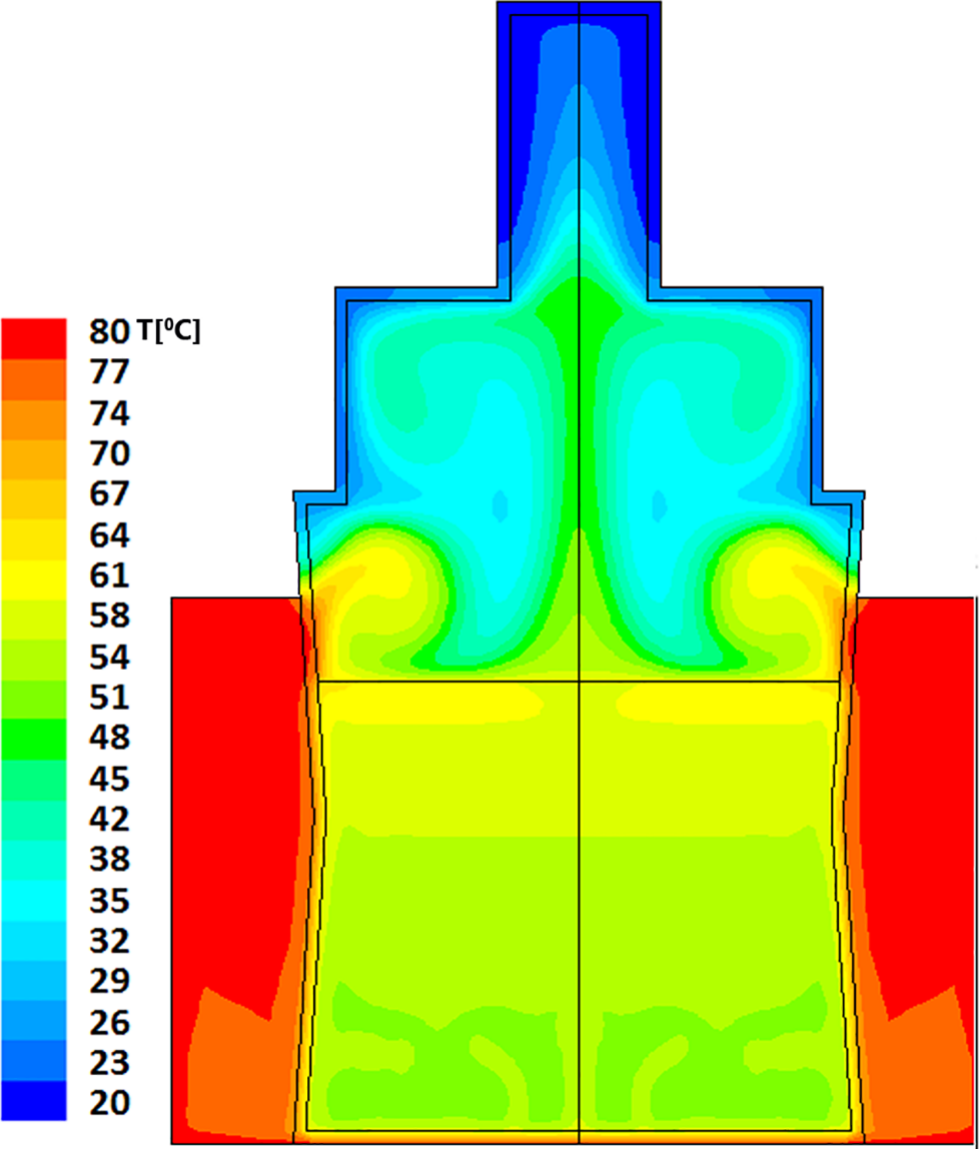
Large volume warming simulation after 600 seconds. The average temperature is 55°C where ~80% of the milk is having temperature of above 58°C. Note that there is also an upper layer of hot milk with temperature above 64°C which is above the temperature of Holder pasteurization (62.5°C). The minimal temperature of the milk is 52°C.

Resultant evolution of temperature profiles within the model bottle-and-heater system are described in Figs 2 and 3 for "Bottle 1" (a model of small (60ml bottle) and Figs 4–6 for "Bottle 2" (large 178ml bottle). As seen in these figures, due to difference in volumes, longer warming periods are needed for large bottles as compared to the smaller ones. Our analysis provides an estimation of percent-milk above maximum-allowed-temperature at different times.

Looking at the time evolution of temperatures within the smaller milk volume, it can be seen (Fig 2) that after 150sec (2.5min) of heating the predicted average temperature (PAT) is already 36°C with 48°C heat zone islets. About 80% of the volume appears to have a temperature below 40°C, the temperature that is considered as the upper desired limit [18]. These higher temperature islets (green-cyan and green) are clearly shown in the upper side of the bottled milk just below the contact with the air where the maximal temperature reaches 48°C. It is clear from this simulation that even after a short warming of 2.5 minutes, ~20% of the milk volume is already at around 40°C and above it. Upon 30 additional seconds of heating (total time 3 minutes) the PAT reaches 40°C with 51°C heat zone islets (data not shown, but available upon request). Further heating for, 4 additional minutes (total of 7 minutes heating time, see Fig 3), yields a very high PAT of 63°C which is a temperature reached during Holder pasteurization. Thus, when heating small volumes of milk, warming of 7 minutes is sufficient to result with hot milk (above 60°C) and not lukewarm milk that is close to body temperature (at around 40°C).

As stated above, larger volumes require longer heating times. The thermal field within the larger bottle, after 5 minutes of heating, is shown in Fig 4; this is the recommended time by the manufacturers for this warning level. The average temperature achieved within the volume of the milk is calculated to be 36°C, though heat islets (green-cyan to green) as high as 53°C are clearly seen in this figure. In this case approximately 25% of the total milk volume has reached temperatures above 40°C. Two additional minutes of heating (total of 7 minutes), yields temperature fields depicted in Fig 5, showing a PAT of 46°C with hot islets (greenish colors) as high as 56°C. Finally, as seen in Fig 6, if the care-giver leaves the bottle in the warmer for 10 minutes, the PAT reaches a value of 55°C with 65°C heat zone islets (yellow).

We also performed (data not seen) calculations of stream-function values for the two cases considered. What is evident is that in both cases multiple flow cells interact in a time-dependent manner thereby leading to strong thermal mixing. In the case of the small volume, the mixing spans most of the volume, while in the case of the larger volume bottle mixing is more concentrated in the lower half of the milk's volume.

It is evident that an important factor for efficiency of heating is the milk bottle. Upright feeders such as the one we used in this simulation are widely used devices to store, warm and feed infant formula or human milk. Upright feeders have been manufactured since the early 20th century (Baby Bottle Museum). Many of the early models were crude instruments made of low quality glass and were mainly of a narrow neck type with pullover nipples. Wide neck upright bottles were manufactured in the UK since the early 1950's and in the USA since the early part of the 20 century. The early US models were of a very wide cylinder type incorporating a large pull over nipple, which simulated the breast. These models later evolved into slightly narrower bottles with a screw thread and retaining ring to hold the nipple in place.

Many of the modern bottles currently sold throughout the world still follow this basic design [23,24]. According to Wikipedia (Wikipedia), the height-to-width ratio of baby bottles is high (relative to adult cups) because it is needed to ensure that the contents flood the teat when used at normal angles, in order to prevent the baby from swallowing air or the fluid from easily tipping. Thus the basic design of baby bottles has not much evolved over the past century. Its shape is feeding-oriented and not warming-oriented and does not have large external surface areas that allow heat exchange and facilitate the heating process. Moreover, the warming fluid cannot be stirred to improve the heating process and to decrease the risk of overheating.

In this study we demonstrated the limitation of commonly used methods of human milk rewarming. It is known that appropriate human milk handling and storage, including warming prior to use, is crucial for preserving its unique properties as well as for avoiding overheating-related accidents. Quantitative understanding of the thermal history of storage and handling procedures is an important step towards the evaluation of existing and planned methods for handling of human milk. We present a computational analysis (i.e. simulations) of heat transfer during the warming of refrigerated human milk using an electric-water based heater and showed that it is difficult to determine when milk reaches the desired temperature. Current methods of rewarming create heat zone islets of high temperatures due to lack of steering. Nevertheless, in most cases, caregivers prefer using the fastest methods that allow feeding the infant as soon as possible without long waiting periods. This choice is in contrast with the period of 20 minutes of steering bottle under lukewarm water environment recommended by the WHO [3].

An additional important issue is not to consider human milk as other hot “drinks”. It is obvious that HM cannot be treated as other hot beverages such as tea, hot chocolate, and coffee that are frequently served at temperatures between 71°C and 85°C with safe optimal drinking temperatures of approximately 58°C [25]. Moreover, a bottle filled with human milk should also not be treated in the same manner as a formula bottle, as human milk is a live fluid. So even temperatures that are considered safe for infant drinking cannot be recommended for the consumption of HM. Parents and health care providers should be aware of these issues especially when sick babies or preterm infants cannot be fed directly at the breast and are bottle or tube fed.

The effect of HM handling procedures was studied mainly due to its relevance to HM banking. However, concerns related to storage and handling are also important for mothers who collect their own milk for later feeding. Container type and shape, amount of air exposure and storage temperature may adversely affect milk stability and composition. recent study [12] questioned the impact of maintaining warmed milk at room temperature. Some studies have identified a significant loss of nutritional and/or immunological properties of the milk, which started at relatively low temperatures. Serial metabolic studies showed that heat treatment of human milk reduces fat absorption by about a third [21,26]. The reduction of fat absorption can be explained on the basis that pasteurization denatures BSSL [27], which seems to takes place at temperatures greater than 40°C [13]. So, when rewarmed at temperatures higher than 40°C (a temperature that most describe subjectively as lukewarm and not hot), human milk nutritional and immunological values begin to deteriorate. At temperatures of 50°C the rate of the milk quality [28] deterioration increases significantly [13]. Studies on pasteurized milk (heated to 62°C for 30 minutes) demonstrated that many immunologic and anti-inflammatory components such as SIgA, lactoferrin, and lysozyme are decreased and beneficial probiotic bacteria and white blood cells are destroyed [28,29].

The benefits of human milk accumulate in a dose response manner: the greater the overall amount of milk an infant receives, the greater the benefit to the infant. The concept of dose response is relevant not only to the percentage of human milk feeds, but also to the duration of human milk feeds over days, weeks and months. Thus the longer an infant receives mother’s milk, the greater the overall benefit [30]. Specifically, studies suggest that the more mother’s milk a preterm infant receives during the NICU stay, the lower the infant’s risks are of NEC, late onset sepsis and enteral feeding intolerance [31–34]. Thus, our findings have significant relevance to NICU preemies. HM has many unique health benefits for the micro-preemie and strategies should be implemented for optimal use of the milk in the NICU [33,35]. Metabolic studies carried out in preterm infants have shown that heat treatment of human milk in terms of reduces fat absorption by about a third [21,26]. This is clearly of importance in the nourishment of preterm infants. Indeed, Preparation of human milk for feedings in NICU may involve some additional processes besides heating that can also alter human milk composition [7,36–39] however, proper heating can prevent excessive heat that in turn can affect the fat profile and enzymes. The loss of fat and lipase activity during treatment is important because fats are the main energy source of breast milk, comprising of 45–55% of the total calories [40,41]. The preparation of human milk in NICU may also affect the fat profiles which has significance as milk secreted by mothers of preterm infants differs in their fat composition from that of mothers of full-term infants. These differences in composition can benefit the preterm infant by providing higher levels of rapidly absorbed fatty acids that are needed for their brain development [42]. Moreover, the preterm infant fat digestion is aided by the bile salt-dependent lipase of human milk [6] which denatures BSSL at around 40°C [27].

## Conclusions

We suggest that there is a need for the development of an efficient but “smarter” method to warm HM to ensure that any milk portion should not exceed 40°C, so its unique properties will be better preserved. Possible solutions may include a mechanism by which human milk would be constantly stirred during heating, or development of novel container that will have large surface area for a narrow milk layer that will enhance the heat exchange and will preserve the unique vivid properties of HM. Such solutions should address the need for fast and safe heating at a reasonable cost that will serve both the home and hospital settings.
