# Correction: Human Milk Warming Temperatures Using a Simulation of Currently Available Storage and Warming Methods

**DOI:** 10.1371/journal.pone.0148970

**Published:** 2016-02-04

**Authors:** Sharron Bransburg-Zabary, Alexander Virozub, Francis B. Mimouni

There is an error in affiliation 1 for author Sharron Bransburg-Zabary. Affilation 1 should be: Nutrits LTD, Tel-Aviv 7239, Israel.

The email address listed for the corresponding author, Dr. Sharron Bransburg-Zabary, is incorrect. The correct email address is: sharron.zabary@gmail.com.

The company “Nutrits LTD” is incorrectly referred to as “nanobébé LTD” in the Funding section. The correct funding information is as follows: Nutrits LTD, provided support in the form of a salary for author SBZ, but did not have any additional role in the study design, data collection and analysis, decision to publish, or preparation of the manuscript.

There are errors in the Competing Interests section. The correct competing interests information is as follows: Dr. Bransburg-Zabary did this study as part of her work in Nutrits LTD and has a patent application title: Container and method for handling and treating a consumable liquid and number: 14/615,407 applied by Nutrits LTD, and the simulations were done as part of preliminary steps of the development of patent name: Container and method for handling and treating a consumable liquid and number: 14/615,407. There are no further patents, products in development, or marketed products to declare. This does not alter the authors' adherence to all the PLOS ONE policies on sharing data and materials.
